# Education Attainment and Obesity: Differential Returns Based on Sexual Orientation

**DOI:** 10.3390/bs9020016

**Published:** 2019-01-29

**Authors:** Shervin Assari

**Affiliations:** 1 Department of Psychiatry, University of Michigan, Ann Arbor, MI 48109-2700, USA; assari@umich.edu; Tel.: +1-(310)-206-5162; Fax: 734-615-8739; 2 Center for Research on Ethnicity, Culture, and Health (CRECH), University of Michigan School of Public Health, Ann Arbor, MI 48109-2029, USA; 3 Department of Psychology, University of California Los Angeles (UCLA), Los Angeles, CA 90095, USA

**Keywords:** population groups, socioeconomic status, education, health behaviors, obesity, body mass index, sexual orientation, sexual minorities, gays, lesbians

## Abstract

***Background:*** Although high educational attainment is linked to better health and lower health risk behaviors, this effect may be systemically smaller for racial and ethnic minority groups compared to Whites. However, it is still unknown whether these diminished returns also apply to marginalization based on sexual orientation. ***Aims:*** In a national sample of adults which was composed of people of color, we compared straight and homosexual people for the association between education attainment and obesity. ***Methods:*** The Social Justice Sexuality Project (SJS-2010) is a cross-sectional national survey of health and wellbeing of predominantly people of color who identify as homosexual. The current analysis included 2884 adults (age 24 or more) who were either heterosexual (n = 260) or homosexual (n = 2624). The predictor variable was education attainment, and the outcome variable was obesity status (body mass index larger than 30 kg/m^2^ [kilograms per meter squared]). Demographic factors (age and gender), household income, nativity (US born vs. immigrant), and health (self-rated health and current smoking) were the covariates. Sexual orientation was the moderator. ***Results:*** In the pooled sample, high education attainment was protective against obesity status. Sexual orientation interacted with education attainment on odds of obesity, which was suggestive of stronger protective effects of high education attainment against obesity for heterosexual than homosexual individuals. ***Conclusion:*** High education attainment better protects heterosexual than homosexual people against obesity, a pattern similar to what has been observed for comparison of Whites and non-Whites. Smaller protective effects of education attainment on health behaviors of marginalized people are possibly, due to prejudice and discrimination that they experience. Discrimination may minimize stigmatized individuals’ abilities to mobilize their economic and human resources and translate them to tangible outcomes. This finding extends the Minorities’ Diminished Returns theory, suggesting that it is not just race/ethnicity but possibly any marginalizing and stigmatizing social identity that results in diminished returns of socioeconomic status resources.

## 1. Background

Minorities’ Diminished Returns (MDR) theory [1,2] suggests that socioeconomic status (SES), particularly education attainment, has a weaker effect on the health and wellbeing of racial and ethnic minority groups compared to Whites. According to this theory, SES difference across racial and ethnic minority groups is not the only cause of racial and ethnic health disparities. Instead, differential health gains of SES indicators partially explain why racial and ethnic health gaps persist, despite considerable efforts toward eliminating them [2]. These diminished returns are, however, traditionally understudied in the field of health disparities.

A considerable body of empirical evidence supports the MDR theory [1,2,3,4]. For example, education attainment better protects Whites than Blacks against obesity [5], self-rated health [6], impulse control [7], sedentary life style [8], poor sleep [8], alcohol consumption [3], diet [9], and smoking [10,11]. Such diminished returns seem to be independent of SES resource, age group, and health outcome. That means, the same patterns can be observed for employment [12,13], income [6,14], marital status [15], quality of neighborhood [16], and size of social network [17] which all better promote the health of Whites relative to Blacks. Almost all of the empirical evidence backing MDR theory is on race / ethnicity, either comparing Blacks [3,4] or Hispanics [11] to Whites.

The smaller health gain of education attainment in racial and ethnic groups compared to Whites can be explained via various economic, social, and psychological mechanisms [1,2]. Structural and institutional discrimination may have a large role [18,19,20,21,22,23,24,25,26,27,28]. Firstly, an increase in education attainment generates more gain in income for Whites than non-Whites [29]. The diminished returns of education attainment on income may be due to the discrimination of non-Whites in the education system or the labor market [30,31,32]. At each education level, Whites have a higher chance of being employed than non-Whites, and if employed, they work in higher paying jobs [33,34]. Job applications are also more successful for Whites than non-Whites, even when the quality of applicants are identical [35]. As a result, far more highly educated non-White than White individuals stay poor [36]. Homosexual individuals may get discriminated against and receive a lower quality of education, have a lower chance of employment. Imprisonment, police shootings, and stop and frisk disproportionately affect people of color [37]. In addition to the low quality of education [38,39,40,41,42,43,44,45,46,47], teachers and principals disproportionately discriminate non-White children, which reduces their education attainment and increase their risk of school dropout [48]. Furthermore, diverse racial and ethnic groups are differently treated by society. As a result, racial and ethnic minority people face more obstacles in their daily lives and to climb the social ladder. As they pay more taxes for the same upward social mobility, they gain less health from it [49,50,51,52]. Psychological processes also play a role in causing diminished returns for racial and ethnic people. Non-Whites encounter discrimination [53], which is a known cause of poor health [54]. Ironically, an increase in SES increases not decreases exposure [55,56] and vulnerability [57,58,59] to discrimination. Among Blacks for example, high SES means a higher likelihood of living in predominantly White neighborhoods, which increases their exposure to discrimination [60,61,62]. As individuals transition from low SES to a high SES, they become more aware of social inequalities and unfairness which is embedded in the social system [63]. High discrimination is shown to minimize the health gains that follow high SES [64]. Thus, upward social mobility might be associated with some deterioration of mental health for non-Whites [65]. For example, at least in some studies, high SES is shown to increase rather than reduce risk of depression for Blacks, particularly Black men [66,67].

Many of these processes may also apply to other marginalized groups who experience discrimination and prejudice. Still, we know more about MDR for race and ethnic groups than other sources of marginalization, such as sexual orientation. While we know that high SES better protects Whites than Blacks from obesity [5], health risk behaviors [8,9], smoking [10,11], alcohol consumption [3], diet [9], breastfeeding and hunger [9], self-rated health [6], impulse control [7], sedentary life style [8], poor sleep [8], poor mental health [14,68,69], poor self-rated health [70,71], chronic disease [72,73], and mortality [74], we do not know if the same differences also exist based on other marginalizing social identities, such as sexual orientation.

## 2. Aim

To expand the literature on MDR theory [1,2] from an exclusive definition of minority status based on race/ethnicity to other forms of marginalizing and stigmatizing social identities, the current study compared the protective effects of education attainment on prevalence of obesity based on sexual orientation [75,76]. In line with previous research documenting stronger protective effects of education attainment against risk of obesity for Whites than Blacks [5], we hypothesized that education attainment would show stronger protective effects against obesity for heterosexual people compared to homosexual individuals. Informed by the MDR theory [1,2], we would expect weaker effects of education attainment against undesired health outcomes among members of marginalized groups which are commonly discriminated against. This is particularly relevant as obesity is more common in homosexual people [77,78,79], homosexual people face high levels of prejudice, bias, discrimination, and hate crimes [80], and education is protective against obesity [5]. 

## 3. Methods

### 3.1. Design and Setting

The current study used data from the Social Justice Sexuality (SJS) project [81,82,83]. Data collection of the SJS project was conducted between January to December 2010 [84,85,86]. Although more information about the SJS project is available elsewhere [84,85,86], we briefly explain the SJS goals and methods here.

### 3.2. Main Study

The SJS project is a large national survey of Black, Latino, Asian and Pacific Islander, and multiracial lesbians, gays, bisexuals, and transgenders (LGBTs). The main purpose of the SJS project was to investigate the experiences of LGBT people of color. Instead of a merely focus on health problems and pathologies, the SJS was designed to capture a more dynamic experience, including happiness, hope, and wellbeing. The study has measurements in the following eight themes: Demographic variables; socioeconomic status; racial identity; sexual identity; gender identity, spirituality/religion; health (mental and physical); family dynamics; political views; and community engagement/volunteer activities. 

### 3.3. Sample and Sampling

The study used convenience sampling. Participants were recruited through multiple data collection strategies and sampling methods, including snowball sampling. Using venue-based sampling, internet-based outreach, partnerships with community-based organizations, as well as activists and opinion leaders, major efforts were made to oversample racial and ethnic groups. The study included a diverse sample by means of age, racial/ethnic identity, sexual orientation, and gender identity. Participants in the SJS project are composed of over 5000 respondents. Participants were sampled from all 50 US states, including Washington DC, and Puerto Rico. Participants were drawn from rural and suburban areas, as well as large urban areas [84,85,86].

### 3.4. Analytical Sample

The current paper is written on a subsample of the SJS participants who were adults (age equal or more than 24 years). As a considerable proportion of SJS sample self-identified as heterosexual, self-identification as homosexual was not considered as an inclusion criterion for our analysis [86,87,88]. This approach enabled us to conceptualize sexual orientation as a moderating variable. The analytical sample was composed of 2884 adults (age 24 or more) who were either heterosexual (n = 260) or homosexual (n = 2624). Homosexual status was defined as identifying either as gay or lesbian. Thus, transgender individuals were excluded from this analysis [83,84,85].

### 3.5. Ethics

All participants provided written consent. The study received ethical approval from the City University of New York (CUNY) institutional review board (IRB approval number = 09-09-1820). 

### 3.6. Data Collection

Participants completed survey instruments which were available in the following two languages: English and Spanish. Study variables included sexual orientation [75,76,87,88,89,90], race/ethnicity, age, gender, education attainment, household income, current smoking, self-rated health (SRH), health insurance, having a regular doctor or healthcare provider, and obesity status.

### 3.7. Dependent Variable

*Obesity Status.* The current study collected data on weight and height of the participants. Individuals self-reported their weight and height. Obesity was defined as a body mass index (BMI) of equal to, or larger than, 30 kg/m^2^, which includes mild, moderate, severe, and morbid obesity [91].

### 3.8. Independent Variable

*Education Attainment.* Educational attainment was measured as below: “1) Less than High School, 2) High School diploma or GED, 3) Some College, no degree, 4) Associates Degree, 5) Bachelor’s Degree, 6) Some Graduate/Professional school, 7) Graduate/Professional degree”. This variable was operationalized as an ordinal variable, ranging from 1 to 7, with a higher score indicating higher education attainment [92].

### 3.9. Confounding Variables

The current study adjusted for several covariates that could be possibly associated with education attainment, as well as obesity. These variables included race/ethnicity, gender, age, household income, smoking status, health insurance, having a regular healthcare provider, and SRH.

*Race/Ethnicity.* Race and ethnicity were self-identified and assessed using the following questions: “Which of the following racial groups comes closest to which you identify (choose all that apply)?” Respondents identified as the following categories: (1) Black, (2) Latino/Hispanic, (3) Asian American/Pacific Islander, (4) Native Americans, (5) Multiracial, (6) Other races, and (7) White. The above categories were coded as follows: (0) White; (1) people of color/Non-Whites (any racial and ethnic groups).

*Demographic factors.* Age and gender (at the time of survey) were measured as demographic confounders. Age was operationalized as a dichotomous variable (25–49 Years versus 50+ Years). Gender was self-reported and a dichotomous variable (female 0, male 1).

*Household Income.* Income was measured using the single item: “Including all income sources, what do you estimate was your total household income last year?” Responses included: (1) under $8500, (2) $8500–$10,999, (3) $11,000–$13,499, (4) $13,500–$14,999, (5) $15,000–$17,499, (6) $17,500–$19,999, (7) $20,000–29,999, (8) $30,000–39,999, (9) $40,000–49,999, (10) $50,000–74,999, (11) $75,000–$99,999, and (12) $100,000 and over. We changed the ordinal categories to an interval measure ranging from 1 to 12. Income of 2 corresponded income between $8500 and $10,999, and income 3 reflected income between $11,000 and $13,499. Household income was treated as an interval variable ranging from 1 to 12, with a higher value indicating higher household income.

*Smoking Status.* Current smoking status was assessed by the following question: “Do you now smoke cigarettes?” Responses included: (1) not at all, (2) some days, and (3) every day. The responses 2 and 3 to this item were used to define a dichotomized variable indicating daily smoking. The same question is used in the Health Information National Trends Survey (HINTS) [93]. This item is a valid and reliable measure in various populations [94,95].

*Self-Rated Health (SRH).* A single item was used to measure SRH. Respondents were asked to rate their overall health as excellent, very good, good, fair, or poor. Literature has shown that SRH is a reliable and valid measure of health [96,97,98,99]. We treated SRH as an ordinal variable, with a higher score indicating worse health status [96,97,98,99]. 

*Health Insurance.* Participants were asked “Do you have health insurance?” The answers included yes (1) and no (0).

*Having a Regular Healthcare Provider.* Participants were questioned “Do you have a regular doctor or health care provider?” The response items included no (0) or yes (1).

### 3.10. Effect Modifier

*Sexual Orientation.* Sexual orientation was self-identified. Participants identified themselves either as homosexual (lesbian/gay) or heterosexual. Individuals were asked “Which one label comes closest to how you describe your sexual identity”. This variable was operationalized as a dichotomous variable: homosexual (1), heterosexual (0) [75,76,87,88,89,100,101].

### 3.11. Statistical Analysis

To analyze the SJS data, we used Survey Documentation Analysis (SDA, University of Michigan, Ann Arbor, MI, United States). For univariate analysis, we described mean and standard errors (SEs) or frequencies, associated with their Confidence Intervals (ICs). We ran four logistic regression models. In all our logistic regression models, education attainment was the independent variable; obesity (BMI ≥ 30) was the dependent variable; sexual orientation was the focal moderator; and race/ethnicity, demographic factors (age and gender), healthcare access (insurance and having a routine healthcare provider), and health status (current smoking and SRH) were the study covariates. The first two logistic regression models were estimated in the pooled sample that included both heterosexual as well as homosexual/LGBT people. The first model only included the main effects and did not include the interaction term. The second model, however, also included the sexual orientation by education attainment interaction term. Subsequently, we performed logistic models specific to groups based on sexual orientation (*Model 3* for heterosexual people and *Model 4* for LGBT people). Odds ratios (OR), SEs, z, as well as *p* values were reported. 

## 4. Results

### 4.1. Descriptive statistics

This study included 2884 adults (age 24 or more) who were either heterosexual (n = 260) or homosexual (n = 2624). Table 1 describes the participants overall and based on sexual orientation. 

### 4.2. Multivariable Models in the Pooled Sample

Table 2 presents the summary of the results of *Model 1* and *Model 2*. These logistic regression models were performed in the pooled sample with education attainment as the independent variable and obesity as the dependent variable. *Model 1* only included the main effect of education attainment and controlled for all other covariates. *Model 2* included the main effects, as well as an interaction term between sexual orientation and education attainment. 

Based on *Model 1*, high education attainment was protective against odds of obesity net of all covariates. *Model 2* revealed a significant interaction between sexual orientation and education attainment on odds of obesity, suggesting that the protective effect of high education attainment against obesity is larger for heterosexual people compared to homosexual individuals (Table 2).

Table 3 summarizes the results of two logistic regressions with education attainment as the independent variable and obesity as the dependent variable in groups defined based on sexual orientation. *Model 3* was performed in heterosexual people and *Model 4* was performed in homosexual individuals. Based on *Model 3* and *Model 4*, high education attainment was protective against obesity for heterosexual individuals, above and beyond covariates. This protective effect, however, was marginally significant for homosexual people. The magnitude of the protective effect of education was also larger for heterosexual than homosexual individuals (Table 3).

## 5. Discussion

Using a national sample of American adults over age of 24 years, this study explored differential returns of education attainment in terms of preventing obesity between homosexual and heterosexual people. Similar to what is previously shown for race and ethnicity [10,11], stigmatizing sexual orientations also reduce the protective effects of education attainment on obesity. Homosexual individuals seem to be in a relative disadvantage compare to heterosexual people for gaining health from their education attainment. 

The protective effect of education attainment against obesity in the overall sample is in line with well-established literatures in the fields of public health, epidemiology, economics, and sociology [102]. The protective effect of education attainment against poor health outcomes is not limited to obesity and is documented for a wide range of health issues [103]. 

The finding of smaller effect of education attainment on obesity status for homosexual compared to heterosexual individuals is in line with previous results for comparison of groups by race and ethnicity [1,2]. Similar patterns are shown for education attainment and other SES indicators on diet [9], alcohol consumption [3], obesity [5], self-rated health [104], impulsivity [7], anxiety [15], and depression [14,68,69]. Multiple studies by Assari [4,13], Hayward [105], Backlund [106], Lewis [107], Roelfs [108], and Everett [109] have shown a larger impact of education attainment on mortality for Whites than non-Whites. Many scholars, such as Navarro [110], Williams [111], Mehta [112], and others [74] have shown that race/ethnicity and SES interact on health, and racial and ethnic group members enjoying smaller health gains deriving as a result of their SES resources compared to the majority group (people with most social and political power). Thus, health disparities are a consequence of nonlinear, complex, inter-related effects between various social identities that shape social status [110,111,112]. 

The current study extends the existing MDR literature from a literature which is exclusively conducted on race/ethnicity to sexual orientation. Although MDR theory is documented across SES indicators, health outcomes, age groups, study designs, and cohorts [1,2], this is the first study to document the same pattern for groups based on their sexual orientation. The results are particularly important as homosexual individuals are at an increased risk of obesity [77,78] and other health problems [113,114]. The results suggest that it is not just lower SES, but the diminished effects of SES that contribute to health disparities [90] in this marginalized population. 

The results of the current study are relevant to the intersectionality framework [115,116,117,118], which suggests life experiences including but not limited to discrimination processes experienced by people are the results of multiplication, not addition, of their stigmatized identities [115,116,119]. In the current study, most participants were non-White homosexual people, with diverse and unique experiences that are different from those of White heterosexual people.

A mechanism that may explain diminished effects of education attainment among stigmatized and marginalized people is the labor market discrimination, that limits the health gains that follow education credentials and years of schooling. Such economic and human resources differently turn to tangible outcomes, such as employment and income, for marginalized and non-marginalized social groups [120,121]. Populations’ ability to translate their education attainment to health is probably mediated by avoiding risk factors and gaining better purchasing power, which is not directly a result of education, but occupation, income, and the prestige that follows education. Such processes widely differ across diverse social groups [122]. Homosexual people may be discriminated against in the job market, particularly if they are open about their sexual orientation, or if they have some phenotypes and behaviors which contrast those expected from their sex/gender. 

This study suggests that what we know about race and ethnicity as a social force that causes diminished returns also applies to other social identities that are associated with stigma and marginalization. That means, like race and ethnic group membership, some sexual orientations may also reduce the returns of SES on health and health behaviors. 

The results of the current study are important for several reasons. First, obesity is a main cause of mortality and mortality in the US and globally [123]. Second, obesity is more common in homosexual that heterosexual individuals [77,78]. Although obesity is under the influence of race/ethnicity [124], SES [124] and sexual orientation [77,78]; these effects are not simple, but complex. Psychological interventions [e.g., cognitive behavioral therapy] can prevent obesity among homosexual individuals [125], however, tailored interventions that consider their marginalized status may be preferred.

The current study contributes to the literature on health disparity among LGBTs [126,127,128,129,130,131,132,133,134,135,136,137,138,139], which is a complex public health matter, similar to disparities in other populations [119,140,141]. The current study leveraged the existing literature by utilizing the MDR theory [1,2] to understand the nontraditional social and structural factors and processes that operate across diverse subgroups. The results suggest that it is not merely lack of access to SES resources or stress, but also diminished returns of available SES resources that contribute to the poor health outcomes of stigmatized groups, broadly defined [75,76,79,87,88,89,125].

## 6. Limitations

This study has some limitations. First, cross-sectional design is not the best design for causal inferences [142,143,144]. Longitudinal studies with multiple observations of SES indicators and BMI are needed to make causal inferences regarding how sexual orientation alters the health effects of education attainment and social mobility. Second, self-reported weight and height was used to measure BMI. Although self-reported data are commonly used to measure BMI in epidemiological studies in the US and worldwide, future investigations may take advantage of direct measurements of height and weight. Third, potential underlying mechanisms are still unknown on why diminished returns of education attainment exist on obesity for homosexual people. One possible mechanism is stress and discrimination. There is also a need to study more SES indicators, including, but not limited to, family type, income, wealth [145,146], employment, and occupation. Finally, due to the small sample size, we could not explore the differences between gays and lesbians. We combined the gay and lesbian people to a single group. Gays and lesbians may, however, differ in their experience of discrimination and prejudice, as well as coping behaviors that may result in obesity. Despite these limitations, the result of the current study extends the existing knowledge on MDR to a broad definition of minority status that goes beyond race and ethnicity and includes sexual orientation. The unique contribution of this study is showing that it is no just race and ethnicity, but probably any marginalizing social identity that can reduce the health gains that are expected to follow SES. 

## 7. Conclusions

To conclude, in line with the literature that has shown race/ethnicity alters the health effects of education attainment, marginalized sexual orientations may also diminish the health returns of education attainment. That is, in America, education attainment better reduces risk of obesity for heterosexual than homosexual individuals. There is a need for social and economic policy implementation that eliminates or at least reduces the effect of diminished returns of education for marginalized people, broadly defined. The policy solutions should go beyond equal access to education and find ways to tackle the many social and environmental obstacles that limit marginalized peoples’ capacity to mobilize their available human and economic resources and turn them into tangible outcomes. There is an urgent need to reduce the existing discrimination against homosexual people across institutions, including, but not limited to, the education system and labor market. Without a drastic decline in the processes involved in marginalization, the very same programs and policies may inadvertently increase rather than decrease the health gap between stigmatized and non- stigmatized groups.

## Figures and Tables

**Table 1:** Descriptive statistics in the overall sample and by sexual orientation (n = 2977).

	All		Heterosexual		Homosexual	
	Mean (SE)	95% CI	Mean (SE)	95% CI	Mean (SE)	95% CI
Self-Rated Health (1–5)	2.33(0.02)	2.30–2.37	2.30(0.06)	2.18–2.42	2.33(0.02)	2.30–2.37
Household Income (0–12)	8.51(0.06)	8.39–8.62	8.07(0.22)	7.64–8.50	8.55(0.06)	8.43–8.67
Education Attainment (0–7)	4.65(0.03)	4.58–4.71	4.52(0.12)	4.29–4.76	4.66(0.04)	4.59–4.73
	**n**	**%**	**n**	**%**	**n**	**%**
Sexual Orientation						
Heterosexual	510	18.07	510	100.00	-	-
Homosexual (Gay/Lesbian)	2312	81.93	-	-	2312	100.00
Person of Color						
No	1727	70.81	47	18.58	551	21.39
Yes	712	29.19	206	81.42	2025	78.61
Gender						
Female	598	21.14	180	73.47	1150	45.33
Male	2231	78.86	65	26.53	1387	54.67
Age						
25–49 Years	1330	47.81	195	79.27	1999	78.24
50+ Years	1452	52.19	51	20.73	556	21.76
Current Smoker						
No	2194	78.33	178	74.79	1810	72.9
Yes	607	21.67	60	25.21	673	27.1
Health Insurance						
No	1988	73.06	55	22.18	486	18.83
Yes	733	26.94	193	77.82	2095	81.17
Having a Regular Doctor/Healthcare Provider						
No	541	19.12	55	22.27	455	17.67
Yes	2288	80.88	192	77.73	2120	82.33
Obese						
No	260	9.02	152	73.79	1575	70.53
Yes	2624	90.98	54	26.21	658	29.47

**Table 2:** Logistic regression models on obesity in the overall sample (n = 2977).

	OR (SE)	95% CI	z	*P*
**Model 1**				
Sexual Orientation (Gay/Lesbian)	1.30 (0.24)	0.91–1.87	1.43	0.153
Race/Ethnicity (non-Whites)	1.18 (0.14)	0.93–1.49	1.34	0.180
Gender (Male)	0.50 (0.05)	0.41–0.61	−6.85	<0.001
Age (50+ Years)	1.42 (0.17)	1.13–1.79	2.96	0.003
Self-Rated Health (1–5)	1.81 (0.10)	1.62–2.03	10.48	<0.001
Smoker (current)	0.72 (0.08)	0.57–0.91	−2.78	0.005
Health Insurance	1.38 (0.22)	1.00–1.90	1.99	0.047
Having a Regular Doctor/Healthcare Provider	0.97 (0.16)	0.70–1.33	−0.20	0.838
Household Income (0–12)	1.02 (0.02)	0.98–1.06	0.95	0.342
Education Attainment (0–7)	0.93 (0.03)	0.87–0.99	−2.32	0.020
Constant	0.05 (0.02)	0.02–0.10	−7.50	<0.001
**Model 2**				
Sexual Orientation (Gay/Lesbian)	0.51 (0.26)	0.19–1.38	−1.33	0.184
Race/Ethnicity (non-Whites)	1.18 (0.14)	0.93–1.49	1.35	0.177
Gender (Male)	0.50 (0.05)	0.41–0.61	−6.86	<0.001
Age (50+ Years)	1.42 (0.17)	1.13–1.79	2.96	0.003
Self-Rated Health (1–5)	1.82 (0.10)	1.63–2.04	10.53	<0.001
Smoker (current)	0.72 (0.08)	0.57–0.91	−2.79	0.005
Health Insurance	1.40 (0.23)	1.02–1.93	2.07	0.038
Having a Regular Doctor/Healthcare Provider	0.95 (0.15)	0.69–1.31	−0.31	0.755
Household Income (0–12)	1.02 (0.02)	0.98–1.06	1.01	0.314
Education Attainment (0–7)	0.77 (0.08)	0.62–0.94	−2.54	0.011
Homosexual × Education Attainment	1.23 (0.13)	1.00–1.52	1.94	0.053
Constant	0.11 (0.06)	0.03–0.34	−3.78	<0.001

Notes. Source: The Social Justice Sexuality Project (SJS-2010); Outcome: Current smoker.

**Table 3:** Summary of logistic regression models based on sexual orientation.

	OR (SE)	95% CI	z	*P*
**Model 3 (Heterosexual)**				
Race/Ethnicity (non-Whites)	1.21 (0.63)	0.43–3.35	0.36	0.721
Gender (Male)	0.70 (0.30)	0.30–1.62	−0.83	0.405
Age (50+ Years)	1.08 (0.53)	0.41–2.84	0.15	0.877
Self-Rated Health (1–5)	2.15 (0.46)	1.42–3.26	3.60	0.000
Smoker (current)	0.52 (0.24)	0.21–1.26	−1.45	0.147
Health Insurance	1.49 (0.94)	0.43–5.12	0.64	0.525
Having a Regular Doctor/Healthcare Provider	0.90 (0.52)	0.29–2.80	−0.18	0.858
Household Income (0–12)	1.00 (0.06)	0.89–1.12	−0.03	0.974
Education Attainment (0–7)	0.76 (0.09)	0.60–0.97	−2.19	0.028
Constant	0.15 (0.22)	0.01–2.51	−1.32	0.187
**Model 4 (Gay/Lesbian)**				
Race/Ethnicity (non-Whites)	1.18 (0.15)	0.92–1.51	1.32	0.187
Gender (Male)	0.49 (0.05)	0.40–0.60	−6.87	<0.001
Age (50+ Years)	1.45 (0.18)	1.14–1.84	3.02	0.003
Self-Rated Health (1–5)	1.80 (0.11)	1.60–2.02	9.88	<0.001
Smoker (current)	0.74 (0.09)	0.58–0.94	−2.49	0.013
Health Insurance	1.39 (0.24)	1.00–1.94	1.97	0.049
Having a Regular Doctor/Healthcare Provider	0.96 (0.16)	0.69–1.34	−0.24	0.808
Household Income (0–12)	1.02 (0.02)	0.98–1.06	0.99	0.323
Education Attainment (0–7)	0.94 (0.03)	0.89–1.01	−1.75	0.080
Constant	0.05 (0.02)	0.02–0.12	−7.30	<0.001

Notes. Source: The Social Justice Sexuality Project (SJS-2010); Outcome: Obesity.
